# Hemodynamic and Pathologic Characterization of the TASK-1^−/−^ Mouse Does Not Demonstrate Pulmonary Hypertension

**DOI:** 10.3389/fmed.2017.00177

**Published:** 2017-10-23

**Authors:** Melanie G. Kitagawa, Julia O. Reynolds, Xander H. T. Wehrens, Robert M. Bryan, Lavannya M. Pandit

**Affiliations:** ^1^Baylor College of Medicine, Texas Children’s Hospital, Houston, TX, United States; ^2^Michael E. DeBakey Veterans Affairs Medical Center, Houston, TX, United States; ^3^Baylor College of Medicine, Houston, TX, United States

**Keywords:** KCNK3, TASK-1, pulmonary hypertension, potassium channels, right ventricular systolic pressures

## Abstract

**Introduction:**

Pulmonary hypertension (PH) carries significant associated morbidity and mortality and the underlying molecular mechanisms of PH are not well understood. Loss-of-function mutations in TASK-1 potassium channels are associated with PH in humans. Although TASK-1 has been considered in the development of PH for over a decade, characterization of TASK-1 knockout mice has been limited to *in vitro* studies or *in vivo* studies in room air at isolated time points. The purpose of this study was twofold. First, we sought to determine if TASK^−/−^ male and female mice developed PH over the span of one year. Second, we sought to determine the effect of chronic hypoxia, a stimulus for PH, and its recovery on PH in TASK-1^−/−^ mice.

**Methods:**

We measured right ventricular systolic pressure (RVSP) and vascular remodeling in male and female C57BL/6 WT and TASK-1^−/−^ mice at separate time points: 20–24 weeks and 1 year of age. Additionally, we measured RVSP and vascular remodeling in TASK-1^−/−^ and wild-type mice between 13 and 16 weeks of age exposed to 10% hypoxia for 3 weeks followed by recovery to room air conditions for an additional 6 weeks.

**Results:**

RVSP was similar between WT and TASK^−/−^ mice. Male and female WT and TASK-1^−/−^ mice all demonstrated age-related increases in RVSP, which correlated to age-related vascular remodeling in male mice but not in female mice. Male TASK-1^−/−^ and WT mice exposed to chronic hypoxia demonstrated increased RVSP, which decreased following room air recovery. WT and TASK-1^−/−^ male mice demonstrated vascular remodeling upon exposure to hypoxia that persisted in room air recovery.

**Conclusion:**

Female and male TASK-1^−/−^ mice do not develop hemodynamic or vascular evidence for PH, but RVSP rises in an age-dependent manner independent of genotype. TASK-1^−/−^ and WT male mice develop hypoxia-induced elevations in RVSP that decrease to baseline after recovery in room air. TASK-1^−/−^ and WT male mice demonstrate vascular remodeling after exposure to hypoxia that persists despite recovery to room air conditions and does not correlate with RVSP normalization.

## Introduction

Pulmonary hypertension (PH) affects both children and adults through various etiologies of disease but with a large associated mortality (1). Features common to all classifications of PH include proliferative remodeling of the vasculature with variable intimal thickening, increased medial thickness, and plexiform lesions in the media of small pulmonary arteries (2). Many questions remain around the molecular basis of PH as well as potential therapeutic strategies that can reverse the pathology (3, 4). One hypothesis surrounding the mechanisms of pulmonary vascular hypercontractility in PH involves inhibition or downregulation of potassium channels in pulmonary artery vascular smooth muscle cells (PAVSMC) (5–7). Inhibition of potassium channel activity would depolarize the cell and increase intracellular Ca^2+^ in PAVSMCs, activating the cellular contractile machinery and subsequent increase in vascular tone (6, 7). Beginning in the early 2000s, several groups have speculated that dysfunction of the two-pore domain potassium channels (K_2P_) could be an underlying mechanism responsible for PH (5, 7–9). A role for the K_2P_ channels in PH remained only speculation until Ma and colleagues reported that loss-of-function mutations in the TWIK-related acid-sensitive K+ channels (TASK; *gene KCNK3*) was identified in connection with familial PH (10). In the affected families, the TASK-1 mutation was inherited in an autosomal dominant manner with incomplete penetrance, as not all family members with the mutation had evidence of disease and the carriers were unaffected.

Previous evidence in animal studies has suggested a role for TASK-1 channels in regulating the resting membrane potential (E_m_) of pulmonary artery vascular smooth muscle, since its voltage-independent gating allows for background conductance of TASK-1 and other K_2P_ channels in vascular smooth muscle cells. This background or “leak” characteristic of these channels enables a hyperpolarized E_m_ (lower voltage) in order to maintain closure of L-type Ca^2+^ channels and maintenance of vasorelaxation (7, 9, 11). Although animal studies have implicated a role for TASK-1 loss-of-function in PH, the overall conclusions of animal studies have yielded inconsistent conclusions and have not demonstrated a definitive role for TASK-1 in PH as compared to the seminal human study. For example, while one group showed long-term pharmacologic inhibition of TASK-1 in rats induced distal neomuscularization and early hemodynamic signs of PH (12), another group demonstrated no difference in resting membrane potential or constrictor responses in TASK-1 knockout mice compared to wild-type controls, suggesting that the TASK-1 does not confer a functional role in pulmonary vascular tone in the mouse (11). More recently, Murtaza and colleagues demonstrated that in isolated ventilated lungs, TASK-1 knockout mice developed hypoxia-induced elevation in pulmonary artery pressures in a comparatively similar fashion to wild-type counterparts (13). While this study highlighted the lack of contribution of the TASK-1 channel to pulmonary vascular tone with acute hypoxia, hemodynamics were not measured in the live animal and, importantly, the response to chronic hypoxia and recovery were not reported. In our current study, we aimed to perform definitive pulmonary hemodynamic and histopathologic characterization of the TASK-1^−/−^ mouse in physiologic chronic hypoxic conditions, as well as recovery in order to ascertain whether or not the TASK-1^−/−^ mouse is a suitable animal model to study PH.

Given the incomplete penetrance of the loss of function of the TASK-1 gene in human PH, we postulated that an additional stimulus is required to manifest the PH phenotype in human disease. The utilization of chronic hypoxia is particularly revealing as hypoxemia is an associated risk factor in the development of certain types of PH and could play an important additional physiologic stimulus for disease along with any genetic contributions of TASK-1 channel dysfunction. In this current study, we applied an additional hypoxic stimulus to the mouse genetic model in order to more closely mimic the physiology of human PH. Importantly, we not only assessed the effect of chronic hypoxia on the TASK^−/−^ genetic background, but also how restoration of room air conditions affected hemodynamic and histopathologic parameters in these TASK^−/−^ mice. Thus, the results of these studies allowed us to fully characterize the TASK-1^−/−^ mice by measuring right ventricular systolic pressures (RVSP) and medial vascular hypertrophy in baseline, hypoxic, and restorative room air conditions in order to clarify the contributions of these channels in the mouse pulmonary vasculature.

## Materials and Methods

### Animals

All studies were approved by the Institutional Animal Care and Use Committee (IACUC) of Baylor College of Medicine. Mice used in these studies were male and female mice at 20–24 weeks of age and 1 year of age. TASK-1^−/−^ (*KCNK3^−/−^*) were a gift from Dr. Bayliss, University of Virginia. TASK-1^−/−^ and wild-type mice were on a C57BL/6 background.

### Right Ventricular Catheterization

Right ventricular systolic pressure, an estimate of pulmonary arterial pressure, was measured in the mice. RVSP was measured in TASK-1^−/−^ and wild-type mice (C57BL/6), one group at 20–24 weeks and another at 1 year of age. Mice were anesthetized with 2% isoflurane in 100% oxygen. A 1.4F high-fidelity micromanometer catheter (Millar Instruments, Houston, TX, USA) was used to measure the RVSP, as previously described (14). RVSP measurements were collected and analyzed with the IOX data acquisition system (Emka Technologies, Falls Church, VA, USA). The mean RVSP was calculated as the average of the maximum pressure measurements after the corresponding minimum pressure measurements were normalized to 0 over at least 15 cardiac cycles and repeated at three different time points. Measurements were confirmed with pressure–volume loop tracings.

### Vessel Histology

Following RVSP measurements, the mice remained anesthetized and their chest was opened to allow for perfusion of the lungs with ice-cold HBSS infused through the right ventricle. The lungs were excised and a portion of the right or left lower lobe was removed and placed in 10% neutral buffered formalin to allow for fixation. The lung was then embedded in paraffin and three 5 μm slices underwent hematoxylin–eosin staining. Images of a minimum of eight vessels about 100 μm in diameter per animal were obtained using a Nikon Eclipse TE2000-U microscope with a 20× objective by a blinded observer. Total vessel area and luminal area were measured using Adobe Photoshop CS3 Extended and ImageJ (NIH). The total vessel area occupied by the vessel wall was measured to determine the thickness of the vessel wall compared to the total vessel size, which was used as an index of wall thickness and a marker for vascular hypertrophy [(total vessel area − luminal area)/(total vessel area) × 100]. The index of vessel wall thickness was measured and averaged over the five vessels for each animal.

### Hypoxic Conditions

TASK-1^−/−^ and wild-type mice (C57BL/6) between 13 and 16 weeks of age were placed in a hypoxia chamber (Biospherix, Ltd.) with 10% oxygen for 3 weeks, a known mechanism to induce PH. Following 3 weeks of hypoxia, a group of TASK-1^−/−^ and wild-type mice underwent RVSP measurements and lung vascular histology assessment as described above. The remaining TASK-1^−/−^ and wild-type mice were transitioned from hypoxia back to 21% oxygen conditions for an additional 6 weeks. Following these 6 weeks of recovery in room air, the remaining mice underwent RVSP measurements and lung vascular histology sampling.

### Statistical Analysis

All data are expressed at mean ± SEM. The data were analyzed with a two-way ANOVA analysis of variance, with Holms–Sidak *post hoc* analysis.

## Results

### Both TASK-1^−/−^ and Wild-Type Mice Demonstrate Age-Related Increases in RVSP

#### RVSP Measurements

**Figure 1:**
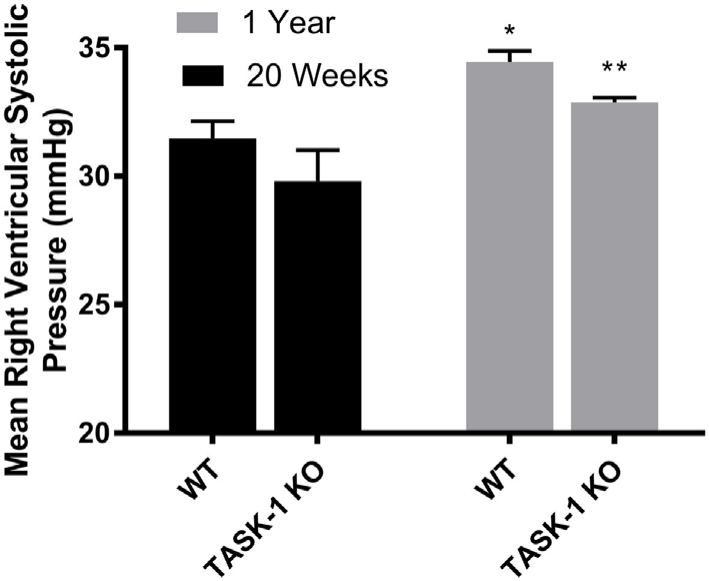
Right ventricular systolic pressures (RVSP) at 20 weeks and 1 year in male TASK-1^−/−^ and wild-type C57BL/6 mice. Wild-type male mice demonstrated an age-related increase in RVSP between 20 weeks and 1 year of age (*p < 0.05, *n* = 5–8). TASK-1^−/−^ male mice also demonstrated an age-related increase in RVSP between 20 weeks and 1 year of age (**p < 0.01, *n* = 5–8 each group).

**Figure 2:**
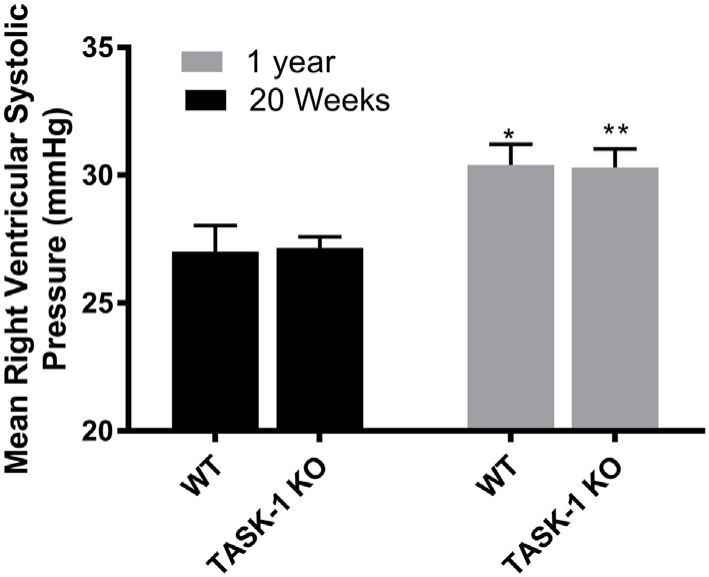
Right ventricular systolic pressures (RVSPs) at 20 weeks and 1 year in female TASK-1^−/−^ and wild-type C57BL/6 mice. Wild-type female mice wild-type demonstrated an age-related increase in RVSP between 20 weeks and 1 year of age (*p < 0.05, *n* = 5–8). TASK-1^−/−^ female mice also demonstrated an age-related increase in RVSP between 20 weeks and 1 year of age (**p < 0.01, *n* = 5–8 each group).

At 20 weeks of age, there were no differences between wild-type and TASK-1^−/−^ (C57BL/6) male mice with regards to RVSP. When RSVP was measured at 1 year of age, male wild-type and TASK-1^−/−^ mice separately demonstrated an age-related increase in RVSP compared to their 20-week-old counterparts (Figure 1). Female wild-type and TASK-1^−/−^ mice similarly did not demonstrate a difference in RVSP at 20 weeks and also showed a separate, age-related elevation of the RVSP from 20 weeks to 1 year of age (Figure 2).

#### Vascular Remodeling in Wild-Type and TASK-1 C57Bl/6 Mice

**Figure 3:**
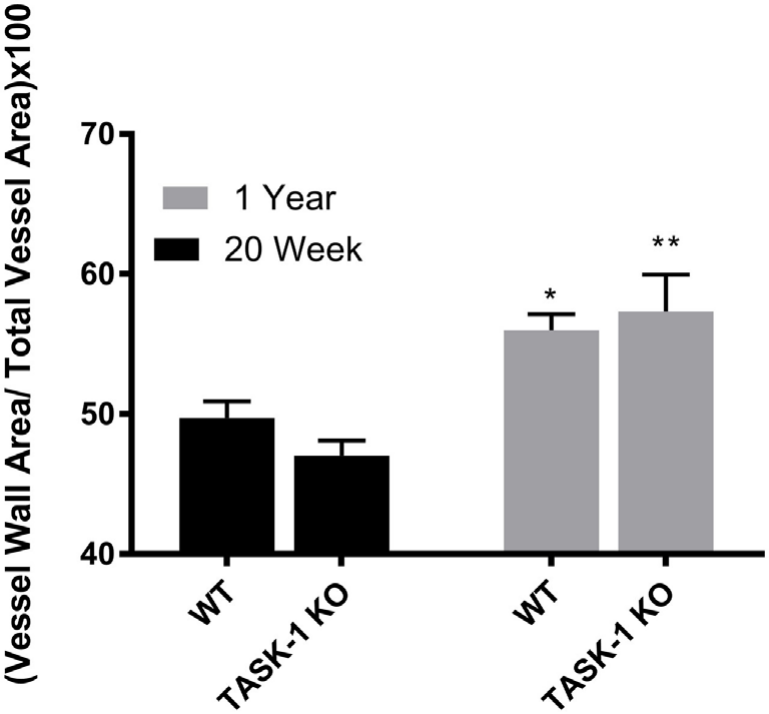
Ratio of medial wall area: total vessel wall area as an index of vessel wall thickness in male TASK-1^−/−^ and wild-type C57BL/6 male mice at 20 weeks and 1 year. While there was no difference in wall thickness index between genotypes, wild-type male mice wild-type demonstrated an age-related increase in wall thickness between 20 weeks and 1 year of age (*p < 0.05, *n* = 5–8). TASK-1^−/−^ male mice also demonstrated an age-related increase in right ventricular systolic pressure between 20 weeks and 1 year of age (**p < 0.01, *n* = 5–8 each group).

**Figure 4:**
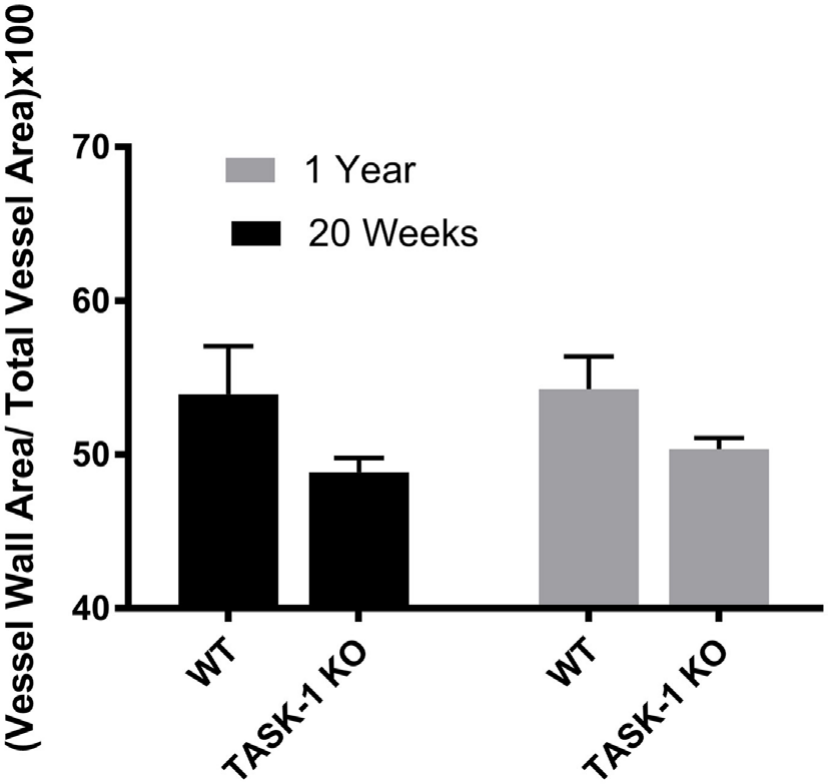
Ratio of medial wall area: total vessel wall area as an index of vessel wall thickness in female TASK-1^−/−^ and wild-type C57BL/6 mice at 20 weeks and 1 year. Female TASK-1^−/−^ mice demonstrated a non-significant trend (*p* = 0.07, *n* = 6–7 animals per group) in decreased wall thickness compared to female wild-type mice at both 20 weeks and 1 year. There was no measurable age-related increase in wall thickness index in either genotype.

Male wild-type and TASK-1^−/−^ mice showed an age-related increase in the ratio of vessel wall area/total vessel area as an index of vessel wall thickness and hypertrophy (Figure 3, *’**p < 0.001) accompanying the increase in pressure. Female TASK-1^−/−^ mice vessel walls also appeared to be show a (non-significant) trend toward a thinner index compared to wild-type 20-week-old mice (*p = 0.07). Moreover, neither female wild-type nor female TASK-1^−/−^ mice demonstrated the age-related increase in vessel wall thickness (Figure 4) that had been observed in the males. The reason for the decreased wall thickness index in the TASK-1^−/−^ and lack of age-related progression in female mice is interesting and discussed further in the manuscript. Thus, in males, RVSP appears to correlate with wall thickness as both increase with age, but in females, this correlation is not observed and thus we surmise that other factors other than vascular pressures likely account for vessel remodeling in female mice.

In summary, although right ventricular pressure measurements were not significantly different between 20-week-old wild-type and TASK^−/−^ mice, hemodynamic measurements did reveal an age-related increase in RVSP in both wild-type and TASK-1^−/−^ mice, and vessel histology in male mice reflected a parallel increase in wall thickness index, which was not observed in female TASK-1^−/−^ mice.

### Hypoxia Exposure Leads to Reversible PH in TASK^−/−^ Male Mice

Since TASK^−/−^ mice (females and males) did not show a difference in mean RVSP or vascular wall thickness compared to wild-type age-matched counterparts, we hypothesized that the addition of hypoxic stimulus to loss of function of the TASK channel would lead to inducible and persistent PH that was otherwise not present with loss of function of the TASK channel alone. This two-hit hypothesis of PH in the TASK channel knockout model would propose that the loss of the channel predisposes to sustained PH once exposed to an additional “hit” from hypoxia.

#### RVSP Measurements

**Figure 5:**
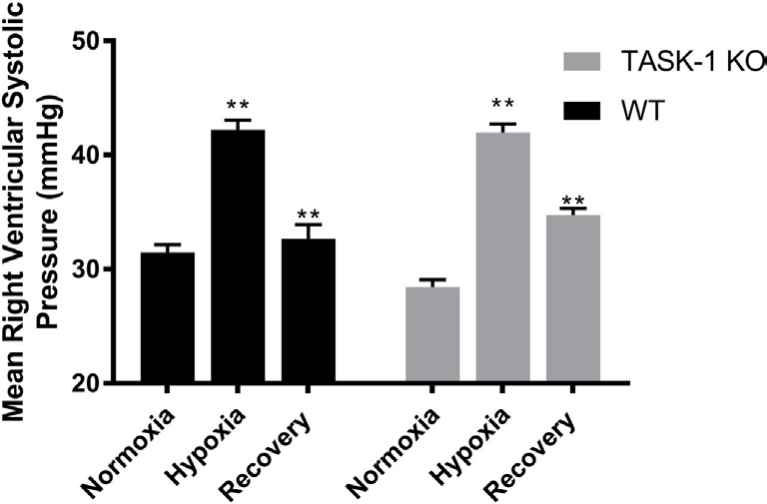
Adult male TASK-1^−/−^ and wild-type mice (C57Bl/6) right ventricular systolic pressures (RVSP) in room air, 10% hypoxia for 3 weeks, then recovery to room air. Wild-type male mice exposed to hypoxia had a significant increase in RVSP hypoxia (*p < 0.05, *n* = 5) from baseline. Hypoxia-induced elevation in RVSP significantly decreased in recovery to room air (**p < 0.001, *n* = 6). TASK-1^−/−^ male mice also demonstrated a similar significant increase in RVSP hypoxia (*p < 0.05, *n* = 6) and that significantly decreased in recovery (**p < 0.01, *n* = 5). There was no difference between wild-type and TASK-1^−/−^ mice in the degree of increase in RVSP after exposure to hypoxia or the recovery decrease in RVSP.

Male wild-type and TASK-1^−/−^ mice exposed to chronic hypoxia demonstrated an elevation of the mean RVSP, which was reversed following the room air recovery period (6 weeks) and decreased back to prehypoxia levels (Figure 5). There were no differences between the genotypes with regards to the degree of hypoxia-induced elevations of RVSP. Thus, both TASK-1^−/−^ and wild-type male mice demonstrated similar increases in RVSP in the presence of chronic hypoxia, which decreased in a similar fashion to almost baseline with a 6-week recovery to room air conditions.

#### Histological Analysis of Vessel Hypertrophy

**Figure 6:**
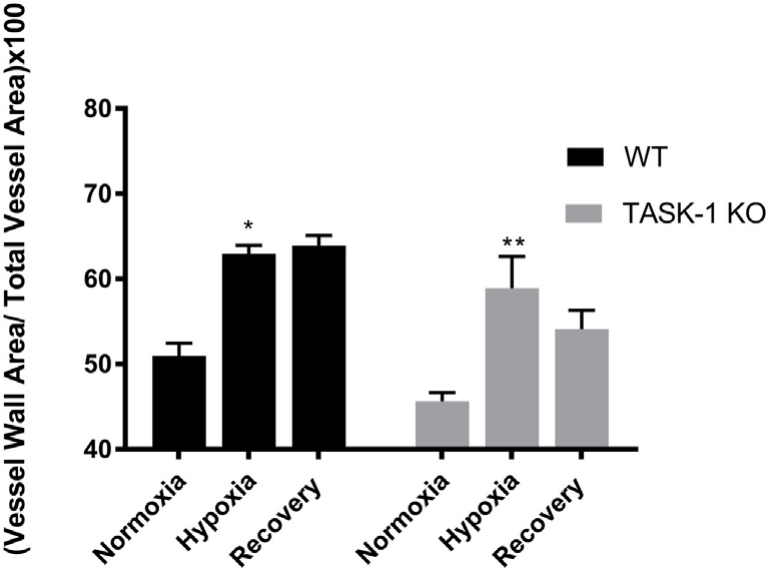
Adult male TASK-1^−/−^ (KO) and wild-type mice (C57Bl/6) index of wall thickness in room air, 10% hypoxia for 3 weeks, then recovery to room air. Both wild-type (*n* = 5) and TASK-1^−/−^ (*n* = 5) animals in hypoxic conditions had increased index of wall thickness compared to baseline normoxia without any difference between genotypes (*p < 0.05, **p < 0.01, wild-type and TASK-1^−/−^, respectively.) The index of wall thickness remained persistently elevated even in recovery in both genotypes.

We examined lung tissue for evidence of pulmonary arteriolar vascular remodeling in TASK-1^−/−^ and wild-type mice (C57BL/6) exposed to chronic hypoxia to determine if the observed increase of RVSP correlated with vascular remodeling (Figure 6). Both wild-type and TASK-1^−/−^ male mice demonstrated a significantly thicker vessel wall index upon exposure to hypoxia (*p < 0.001, **p < 0.01). Similar to hypoxia-induced elevations in RVSP (Figure 5), there were no genotype-related differences within hypoxia or recovery conditions. Interestingly, the increased wall thickness observed with chronic hypoxia in both genotypes persisted even after recovery to room air in both genotypes. We discuss these observations below.

## Discussion

The K_2P_ channel TASK-1 has been implicated in PH in human studies, but recent mouse studies have failed to show a clear role for TASK-1 in the development of PH. In this study, we extended our understanding of the role of the TASK-1 potassium channels in the mouse pulmonary vasculature and their role in PH. Our results demonstrated that (i) neither female nor male TASK-1^−/−^ (C57BL/6) mice develop hemodynamic or vascular evidence for PH at 20 weeks, but that RVSP rises in an age dependent manner in both genotypes to a similar degree; (ii) while wild-type and TASK-1^−/−^ male mice demonstrate age-related increased vascular wall thickness and remodeling that parallel the age-related increase in RVSP, female and wild-type and TASK-1^−/−^ mice do not develop age-related vascular wall thickness or evidence of remodeling as they age; (iii) TASK-1^−/−^ and wild-type (C57BL/6) male mice both develop hemodynamic and vascular evidence for PH to similar degrees in the presence of 10% hypoxia, but this phenotype is not sustained hemodynamically as pulmonary pressure decrease to baseline pre-hypoxia levels after recovery period; (iv) TASK-1^−/−^ and wild-type (C57BL/6) male mice demonstrate vascular remodeling after exposure to 10% hypoxia that appears to be persistent despite 6 weeks of recovery to room air conditions and does not correlate with a normalization of pulmonary pressures.

Ma and colleagues initially reported that loss-of-function mutations in the KCNK3 gene encoding the TASK-1 potassium channels channel were identified in connection with familial PH (10). The parallel animal studies have suggested a role for TASK-1 channels in regulating the resting membrane potential (Em) of pulmonary artery vascular smooth muscle. However, the role of TASK-1 in setting pulmonary vascular tone is yet unknown as the human study demonstrated incomplete penetrance of the mutation within a family and the animal models have not been fully phenotypically characterized. While genetically modified mouse models of PH are powerful tools that can be utilized to isolate and study specific molecular pathways leading to disease, interpreting the results of PH experiments in mice cannot be broadly applied to human disease (15). PH is a disease of diverse etiologies that cannot be studied by the presence of merely one genetic variant. This study highlights the utility as well as drawbacks of a TASK^−/−^ mouse in order to study the role of this member of K_2P_ family of ion channels in the development of PH. While loss-of-function mutations in the TASK-1 has been confirmed as instrumental in the development of hereditable forms of human PH, the K_2P_ channels have not been consistently shown to play a well-defined role in the mouse model of disease. Certainly, characterizing any mouse model of disease requires comparison to a “gold standard,” and this aspect is challenging in pulmonary vascular research since published literature demonstrates a wide spectrum of values and measurements in defining the presence of PH as our work and others have shown (6, 14–16). Moreover, in human PH, pulmonary pressures (and right ventricular pressure overload) and the degree of hypoxia do not consistently correlate with the degree of vascular remodeling until late in the progression of disease (17, 18).

Our study showed that both female and male TASK-1^−/−^ mice did not develop elevated RVSP and one possible mechanism could be compensatory expression of other K_2P_ family genes, specifically the TREK-1 channel (KCNK2) in TASK-1^−/−^ mice (see Figure S1 in Supplementary Material), particularly given the role of the multiple K2P channels as background “leak” channels expressed in the pulmonary vascular system (7, 9) Our study also revealed aging-related findings similar to human data, in that all mice of both genders (male and female), in all genotypes (wild-type and TASK^−/−^) reflected age-related increases in right ventricular systolic pressures (RVSP). These increases in RVSP correlated to increases in vascular wall thickness in male, but not in female mice. This lack of correlation between RVSP and vasculopathy could reflect the stage of reversibility in PH and related vasculopathy that our study animals represent. Estrogen effects on vascular disease have been well described and a recent *in vitro* rats study demonstrated that estradiol stimulation of the smooth muscle estrogen receptor vascular smooth cells significantly attenuated the extent of medial hypertrophy (19). Pathology studies in lung tissues from PH patients have shown that the disease progresses from a reversible vasculopathy characterized by medial hypertrophy to a more irreversible form of disease with intimal proliferation and a more robust vasculopathy. Moreover, others have corroborated our age-related findings and have shown that the developmental stage of the organism greatly modifies the response of the pulmonary circulation to injury (17, 18, 20). Our findings in this TASK^−/−^ mouse model may represent a previously unreported progressive and malleable, age-related change in hemodynamics of the TASK-1^−/−^ mouse.

Hypoxia is a known stimulus in the development of pulmonary vasoconstriction and subsequent vascular remodeling leading to increased pulmonary vascular resistance. While we have limited data on the effect of physiologic hypoxia on TASK-1 expression in patients with PH, an elegant study has shown that TASK-1 channel expression is sensitive to acute hypoxia in primary pulmonary vascular smooth muscle cells from healthy patients (21). We attempted to characterize the phenotype of physiologically (hypoxia) induced PH in the TASK-1^−/−^ mouse in order to measure this potential interaction *in vivo*. The hypoxia studies described here demonstrate how hypoxia-induced RVSP elevation in male mice correlate to vasculopathy with no differences observed between TASK-1^−/−^ and wild-type mice. While we demonstrated that hypoxia-induced PH was equally reversible in both the TASK-1^−/−^ and wild-type male to almost baseline levels, the increased wall thickness persisted despite recovery to room air conditions. The lack of attenuation of vascular remodeling despite restoration to room air, and the lack of correlation to RVSP attenuation in both wild-type and TASK-1^−/−^ animals is interesting and points to other factors other than increased pressure as a determinant of remodeling. Certainly, a longer recovery period beyond 6 weeks may restore the vascular medial hypertrophy, but clearly pressure alterations alone do not determine pulmonary vasculopathy in mice. Pulmonary vascular remodeling involves all layers of the vessel wall and is complicated by the finding that cellular heterogeneity exists within the vascular wall: intima, media, and adventitia. While studies have described hypoxia interactions with K^+^ channels to alter vascular smooth muscle cell contraction, it also regulates vascular wall cell proliferation and apoptosis, which also contribute to remodeling apart from increases in pressure alone (6, 8, 17).

Like other K_2P_ family members, the TASK-1 channel’s role as a “leak” channel would suggest that it contributes to the resting membrane potential (Em) in PASMCs. It may be then surmised that the Em resting potential of PASMCs from TASK-1^−/−^ mice would be comparatively depolarized. However, Manoury and colleagues have already demonstrated that current clamp recordings confirmed no differences in Em in PASMCs from WT and TASK-1/3 KO mice (11). They concluded that there is no evidence that TASK-1 channels contribute to the background K+ conductance or resting Em in the mouse pulmonary artery. We also demonstrated no difference in Em between WT and TASK-1^−/−^ mice PASMCS (Figure S2 in Supplementary Material). We surmise that that although the role of TASK-1 as an isolated channel may not be sufficient to impact vascular tone and remodeling, the contribution of combined K2P channels plays an as yet, undiscovered role. Certainly future characterization studies in K2P channel genetic double genetic knockout mice may offer more information on the individual channel roles.

Different K_2P_ channels may play variable contributory roles to pulmonary vascular tone in human disease. TASK-1 mutations have been implicated in certain forms of hereditable PH but certainly cannot explain all forms of PH, particularly when the disease is caused in part by hypoxia or heart disease. This study augments our current knowledge that various contributing factors, genetic and environmental, combine to cause a vascular phenotype in PH (22). Further studies in human and mice models of PH are required at a cellular and vascular level to discern the individual contributions of the K_2P_ channels. Experimental manipulation of physiologic conditions in animal models can enhance our understanding in how environmental changes in human PH can combine with genome-line changes to cause disease. We can harness the knowledge of these environmental effects and apply it to modified mouse models to determine the mechanisms behind the physiologic processes of human PH.

## Ethics Statement

All studies were approved by the Institutional Animal Care and Use Committee (IACUC) of Baylor College of Medicine. Mice used in these studies were male and female mice at 20–24 weeks of age and 1 year of age.

## Author Contributions

MK and JR performed all of the experiments described in the manuscript, prepared the figures, and wrote the methodology. RB and LP designed the experiments and performed the statistical analysis on the data. LP, RB, and XW provided technical and laboratory support for the research as well as manuscript preparation.

## Conflict of Interest Statement

The authors declare that the research was conducted in the absence of any commercial or financial relationships that could be construed as a potential conflict of interest.
