# Supplementary material for: Hemodynamic and Pathologic Characterization of the TASK-1−/− Mouse Does Not Demonstrate Pulmonary Hypertension
Source: Front Med (Lausanne). 2017 Oct 23;4:177. doi: 10.3389/fmed.2017.00177 (PMC5660113; doi:10.3389/fmed.2017.00177)
Supplement: Supplementary file 1 [file Data_Sheet_1.DOCX]

Supplemental Methods:

RNA isolation and real-time polymerase chain reaction (RT-PCR): RNA was isolated from the whole lung and pulmonary artery from TASK-1 -/- and wild-type (WT) male mice (20 weeks of age). The mice were anesthetized with a cocktail of ketamine (37.5 mg/ml)-xylazine (1.9 mg/ml)-acepromazine (0.7 mg/ml) followed by administration of heparin (50 units, McKesson). Under sterile conditions, the thoracic cavity was opened and the pulmonary vasculature was flushed with ice-cold Hanks’ Balanced Salt Solution 1X (HBSS, Gibco) via cardiopuncture. The lung was removed, separated from the pulmonary artery, and immediately flash frozen. The pulmonary artery and the second order branches were placed in ice-cold HBSS, cleaned of connective tissue and fat, and flash frozen. At a later date, the frozen lungs were homogenized in TRIzol LS reagent (Invitrogen). RNA was extracted from both the pulmonary artery and the lung homogenate using RNeasy Micro kit (QIAGEN) according to the manufacturer’s instruction. The lungs were additionally treated with DNase (Invitrogen). RNA purity was assessed by nanodrop spectrophotometry with the acceptable 260/280 absorbance ratio being > 1.9. Reverse transcription was conducted on 0.1 μg and 1 μg total RNA for the pulmonary artery and lung respectively using random hexamers and Superscript III. Primers were designed using Primer Express 2.0 software and listed below. The gene abbreviation is followed by the NCBI accession number and amplicon size: Gapdh [NM_008084 (111 nt)], fwd 5’-AGCCTCGTCCCGTAGACAAAA and rev 5’-TGGCAACAATCTCCACTTTGC; TASK-1 (K2P3.1) [NM_010608 (135 nt)], fwd 5’-ACATGGACTCCCCTTTGCTGT and rev 5’-CAAATGAATACGGAGGTGGCA; TWIK-2 (K2P6.1)[NM_001033525 (119 nt)], fwd 5’-AGGCATCGAAACCAGACGTGT and rev 5’-TCCCCCGTGTGACTTTCTACA.

Electrophysiologic Studies: The secondary branches of pulmonary arteries from 20 week-old TASK-1-/- and wildtype male mice were dissected and minced into 1 mm pieces followed by enzymatic digestion with a papain/dithiothreitol cocktail, followed by a collagenase H /hyaluronidase cocktail (Sigma). The mixture is then triturated to form a suspension of individual PASMCs. The cells are plated and identified on a coverslip (10-60 cells/coverslip). Membrane potential (Em) was determined using current clamp (I) in the whole cellmode. All readings were measured using an Axopatch 200B integrated patch clamp amplifier and pClamp version 9.2 software.

SUPPLEMENTAL FIGURE LEGENDS:

S1. mRNA expression of K2P channels in extracted pulmonary artery from wildtype and TASK-1-/- male (C57BL/6) mice. (*p<0.05, n=4 each group)

S2. Resting membrane potential in freshly dispersed pulmonary artery smooth muscle cells from wildtype and TASK-1-/- male (C57BL/6) mice. (n=5 animals per group, 5-8 cells per animals)
