# Supplementary figures and images for: Hemodynamic and Pathologic Characterization of the TASK-1−/− Mouse Does Not Demonstrate Pulmonary Hypertension
Source: Front Med (Lausanne). 2017 Oct 23;4:177. doi: 10.3389/fmed.2017.00177 (PMC5660113; doi:10.3389/fmed.2017.00177)

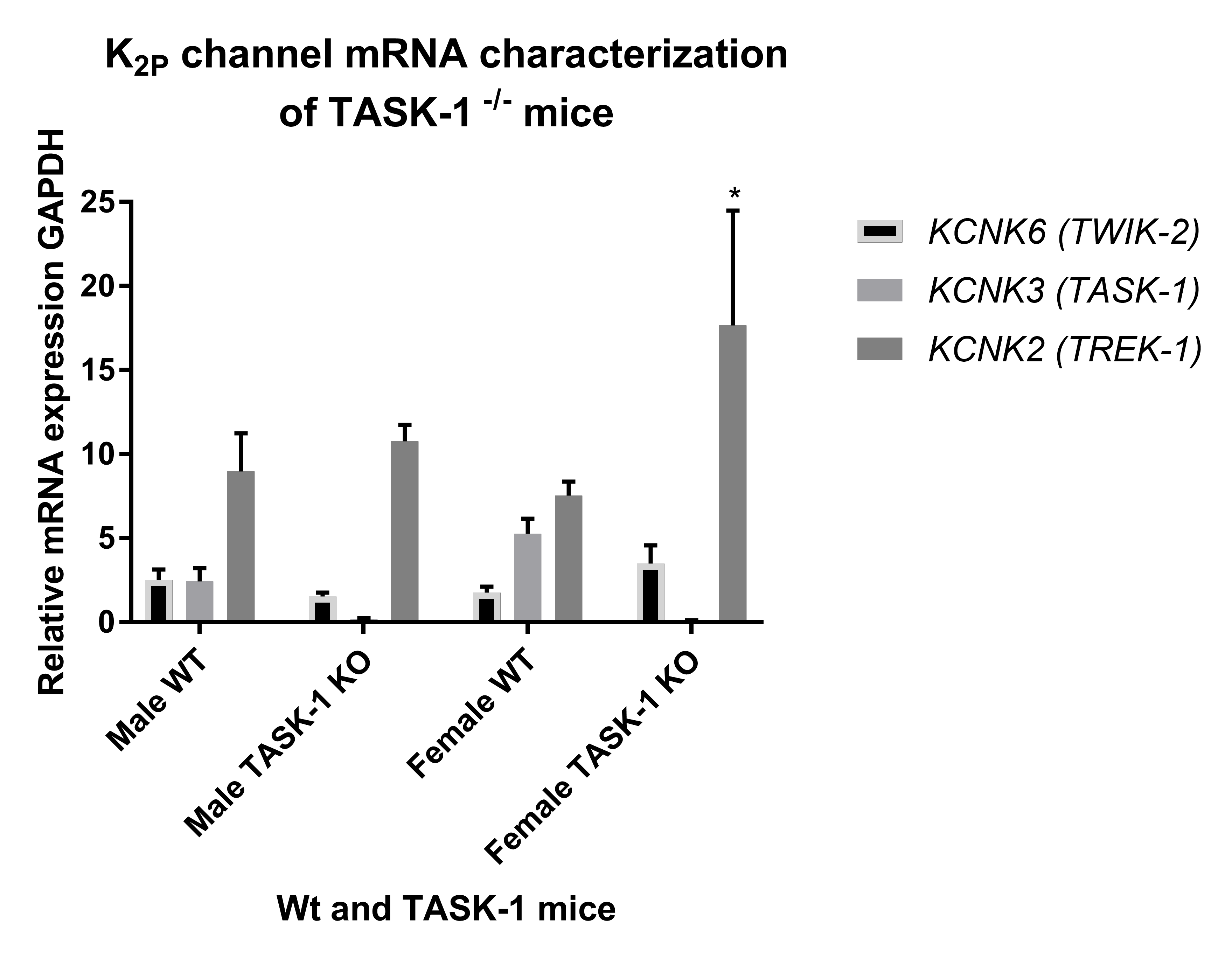

Supplement: Supplementary file 2 [file Image_1.TIF]

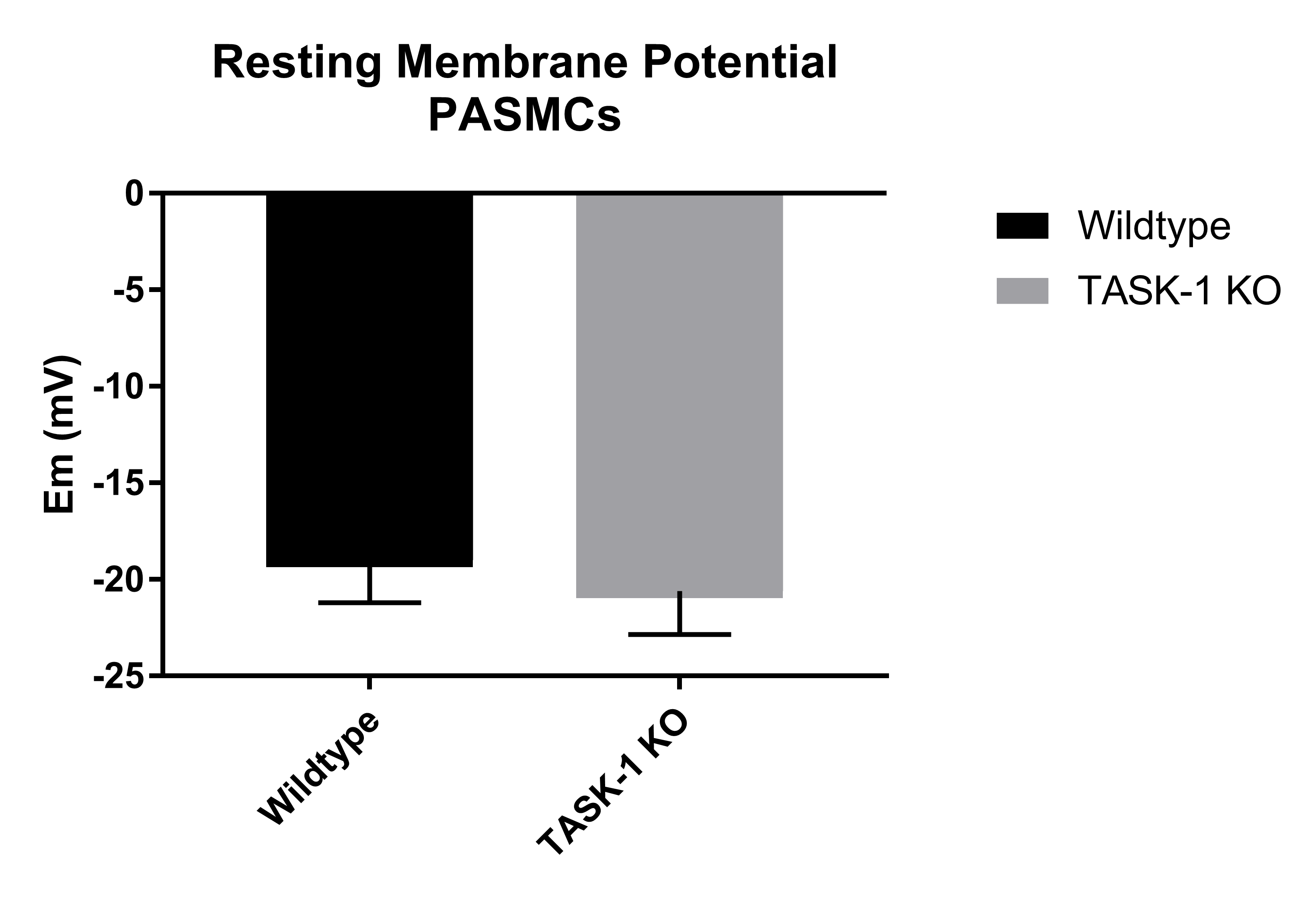

Supplement: Supplementary file 3 [file Image_2.TIF]
